# En bloc kidney transplantation from pediatric donors to teenage recipients: Two case reports

**DOI:** 10.1002/iju5.12686

**Published:** 2023-12-27

**Authors:** Takafumi Yagisawa, Taichi Kanzawa, Toshihito Hirai, Kohei Unagami, Yoko Shirai, Kiyonobu Ishizuka, Kenichiro Miura, Motoshi Hattori, Hideki Ishida, Toshio Takagi

**Affiliations:** ^1^ Department of Urology Tokyo Women's Medical University Tokyo Japan; ^2^ Department of Organ Transplantation Tokyo Women's Medical University Tokyo Japan; ^3^ Department of Pediatric Nephrology Tokyo Women's Medical University Tokyo Japan

**Keywords:** deceased donor, en bloc transplant, kidney transplantation, pediatric donor

## Abstract

**Introduction:**

Since the implementation of the new selection criteria in 2018, kidney donations from pediatric patients have been prioritized for pediatric recipients and kidney donations from pediatric donors have increased in Japan. Herein, we present two cases of en bloc kidney transplantation.

**Case presentation:**

Case 1: A 19‐year‐old male patient who had been on hemodialysis for 5 years due to end‐stage renal disease. After brain death, a graft from a 5‐year‐old boy was transplanted into the right iliac fossa. Case 2: A 19‐year‐old male patient, who had previously undergone a living kidney transplantation at the age of 3, received a secondary cadaveric kidney transplantation in the left iliac fossa. The graft was procured from a 17‐month‐old girl following cardiac death.

**Conclusion:**

This report will help surgeons perform en bloc kidney transplantation in the growing number of pediatric kidney donations, such as those in Japan.

**Abbreviations & Acronyms**
AA: abdominal aorta
CT: computed tomography
EBKT: en bloc kidney transplantation
EIA: external iliac artery
EIV: external iliac vein
ESRD: end‐stage renal disease
IIA: internal iliac artery
IVC: inferior vena cava
LRKT: living‐related kidney transplantation
POD: postoperative day
POM: postoperative month
SCr: serum creatinine
TIT: total ischemic time
WIT: warm ischemic time

**Keynote message**

In the present report, we describe the surgical experiences and short‐term postoperative period with stable renal function in adolescent recipients of en bloc kidney transplantation. This report provides supporting evidence for using en bloc kidney grafts in adolescent recipients.

## Introduction

Many countries worldwide have a variety of priorities in kidney donation programs for children with ESRD to give them the opportunity to receive a kidney transplant sooner.^1^, ^2^ Since the revised Organ Transplant Law came into effect in 2011 in Japan, 10–25 pediatric cadaveric kidney transplants have been performed annually.^3^ Pediatric kidney donations have increased since the announcement of the new selection criteria in 2018, which stated that pediatric recipients are acquired priority to receive kidneys from pediatric donors. Donor kidneys from underweight pediatric patients are recommended for EBKT to prevent complications, such as over‐filtration into the glomerulus.^4^, ^5^ The concept of EBKT in clinical practice was first described in the 1960s.^6^ In recent years, studies have reported that the graft function of EBKT is better than that of standard kidney transplantation in the adult recipient cohort.^7^ However, there are not enough reports on EBKT in children. Herein, we report two cases of EBKT from pediatric donors to teenage recipients performed at our institution.

## Case presentation

### Case 1

**Fig. 1:**
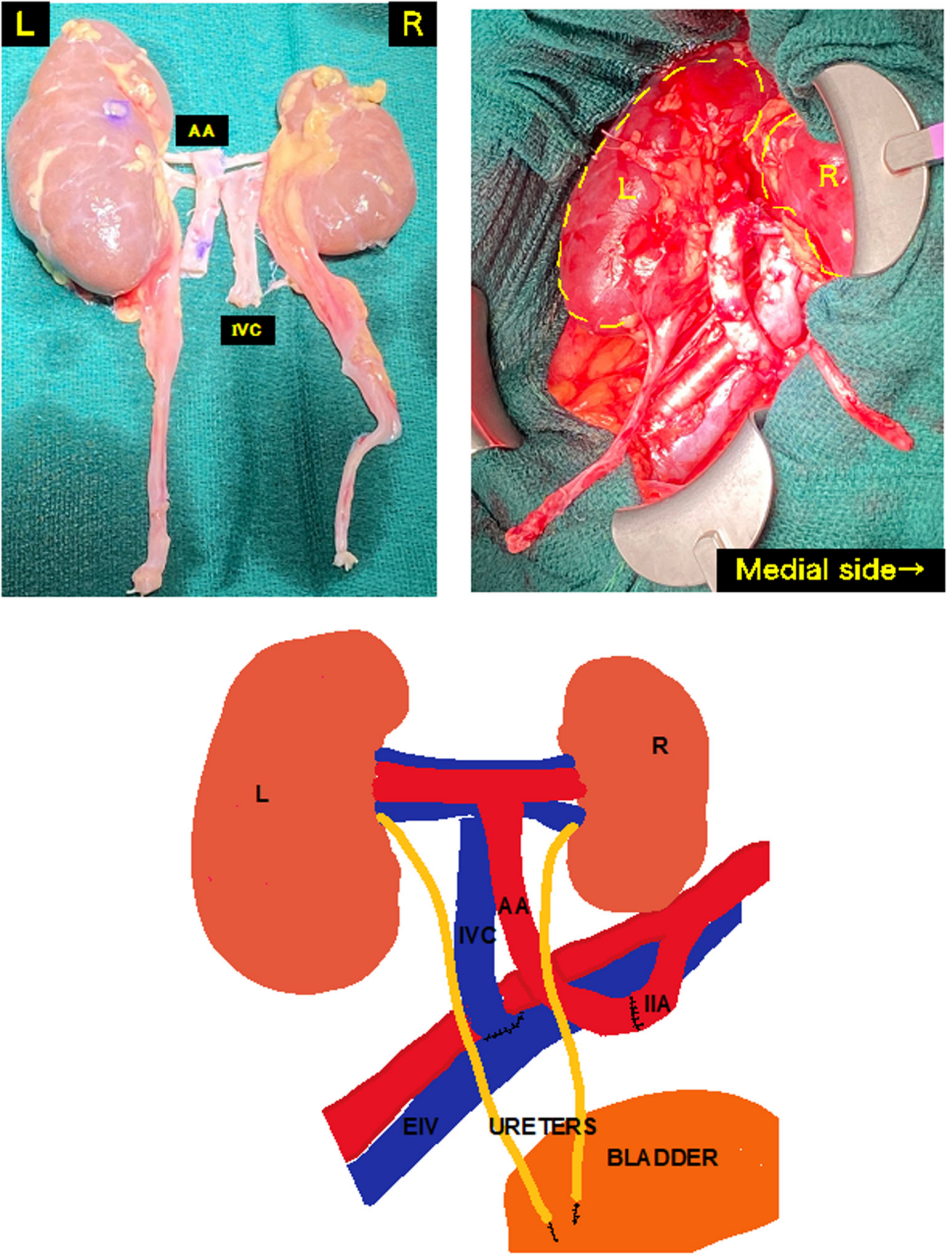
Case 1: En bloc kidney graft was transplanted into the right iliac fossa. End‐to‐end anastomosis of the en bloc graft's aorta to the IIA and end‐to‐side anastomosis of the en bloc graft's IVC to the EIV.

**Fig. 2:**
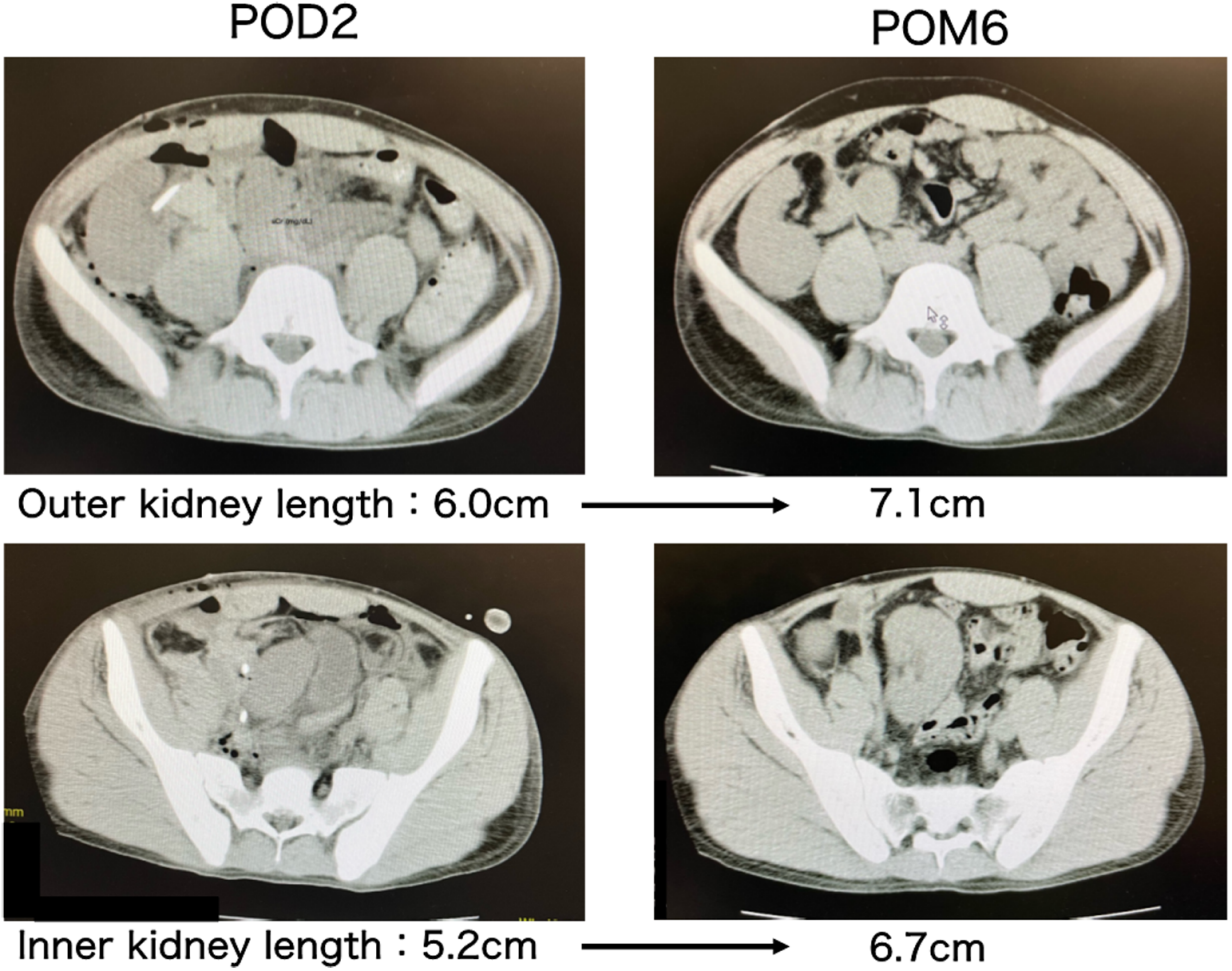
Case 1: Changes in each kidney length during the postoperative period. Both kidneys were growing during the posttransplant period.

A 19‐year‐old male with ESRD due to Alport syndrome, with a height of 173 cm and weight of 57 kg, had been on hemodialysis for 4 years. The donor after brain death was a 5‐year‐old, 110 cm tall, and weighed 15.3 kg male patient. The SCr level was 0.23 mg/dL. The left kidney was significantly larger than the right kidney (Fig. 1). Immunosuppressants, such as tacrolimus, mycophenolate mofetil, methylprednisolone, and basiliximab, were administered. The en bloc graft flipped 180° and was transplanted into the right iliac fossa of the recipient. The distal end of the graft's IVC was anastomosed to the EIV in an end‐to‐side manner using 5‐0 Prolene. The distal end of the graft's AA was anastomosed to the IIA in an end‐to‐end manner a using 5‐0 Prolene suture. Extravesicular anastomosis (Lich‐Gregoir technique) was performed in each ureter with a 4‐0 Maxson suture and performed with a 4.7 Fr stent placement. The two ureters were individually anastomosed to the bladder. The passage of the first drop of urine was observed 25 min after reperfusion. The operative time was 5 h and 22 min, and the TIT was 11 h and 11 min. The SCr level decreased from 10.5 before transplantation to 0.84 mg/dL 4 weeks after transplantation. The ureteral stents were removed at 4 weeks postoperatively. After 1 year of follow‐up, the SCr level was approximately 0.9 mg/dL. The longest diameter of the kidney was measured at each time point (Fig. 2). Each kidney grew significantly in half a year.

### Case 2

**Fig. 3:**
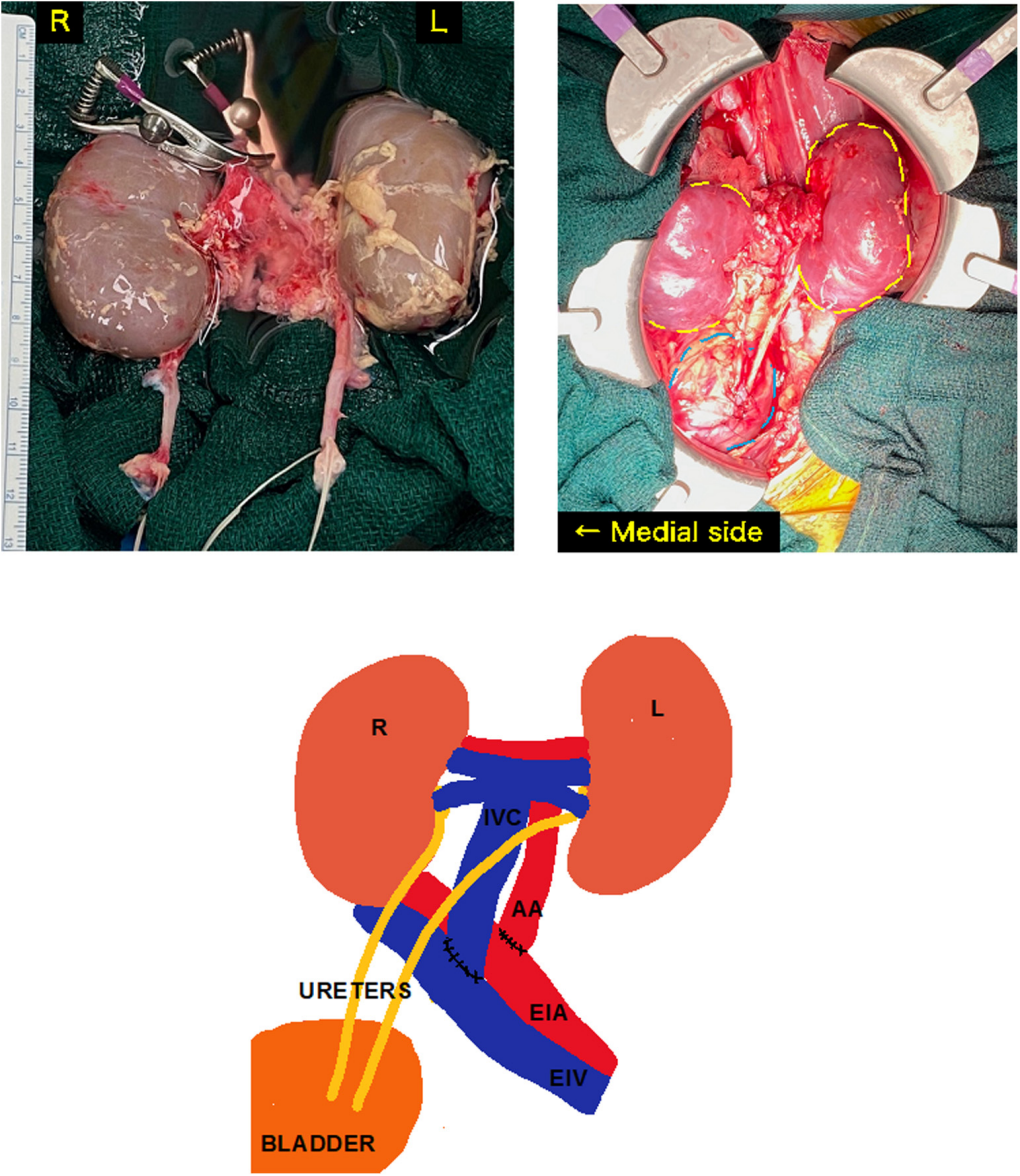
Case 2: En bloc kidney graft was transplanted into the left iliac fossa. End‐to‐side anastomosis of the en bloc graft's aorta to the EIA and end‐to‐side anastomosis of the en bloc graft's IVC to the EIV.

**Fig. 4:**
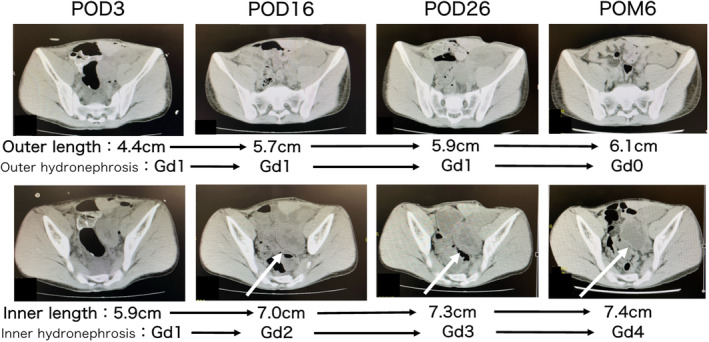
Case 2: Variation of each kidney length and hydronephrosis grading according to the postoperative period. The upper figures show the outer kidney and the lower figures show the inner one. The renal parenchyma of the inner kidney was considerably thinned due to hydronephrosis 6 months after the transplantation. Hydronephrosis grading is based on the Society for Fetal Urology (SFU) grading system.

A 19‐year‐old male who was 168 cm tall and weighed 55 kg. He had undergone LRKT at the age of 3 in the right iliac fossa due to a hypoplastic kidney. Unfortunately, rejection results in graft loss 16 years posttransplantation. The donor after circulatory death was a 17‐month‐old, 6 kg female patient. The distal end of the AA of the graft was anastomosed to the side of the EIA, and the distal end of the graft's IVC was anastomosed to the side of the EIV using a 5‐0 Prolene suture (Fig. 3). The ureters were anastomosed separately same as Case 1, without ureteral stents. The TIT and WIT were 725 and 30 min, respectively. The operative times were 3 h and 37 min. The immunosuppressant agents administered were the same as in Case 1. SCr decreased from 7.08 to 1.99 mg/dL 4 weeks posttransplant. SCr remained at 1.8 to 2.0 mg/dL 10 months after surgery. Although both kidneys grew over time, hydronephrosis was observed on the inner side of the kidney (Fig. 4). To address this, conservative management was done. This decision was made based on the patient's favorable postoperative renal function.

## Discussion

We decided to perform EBKT from a 5‐year‐old donor to a 19‐year‐old recipient and a 17‐month‐old donor to a 19‐year‐old recipient. Currently, there are no clear criteria for a single or en bloc in transplant in pediatric recipients. Kayler *et al*. reported that kidneys from pediatric donors weighing less than 10 kg are generally more safely transplanted as en bloc.^8^ They, along with other researchers, noted a critical threshold for donors weighing 15–20 kg that influences the choice between single and en bloc transplantation.^8^, ^9^ In our experience, the donor weight was approximately 16 kg in Case 1 and <10 kg in Case 2. In Case 1, the donor's left and right kidneys exhibited a significant size discrepancy, with the right kidney notably smaller. An en bloc transplantation was chosen for this case (Fig. 1).

In assessing posttransplant kidney growth, we measured kidney length at various time points. In both cases, there was a gradual bilateral increase in size of the transplanted kidneys up to 6 months postoperatively. Regarding kidney growth posttransplant, Feltran *et al*. reported that kidneys from donors aged 16 and below have a tendency to increase over time following transplantation, accompanied by mild compensatory hypertrophy of nephrons.^10^

Transplant surgeons need to take care of ureteral complications such as ureteral stenosis and leakage from the vesicoureteral anastomosis. In our experience, we retrieved the ureters separately during procurement. Although both recipients were approximately 170 cm in length, the ureters of the 5‐year‐old 15 kg donor in Case 1 and the 17‐month‐old 6 kg donor in Case 2 were sufficient to reach the bladder. We performed the vascular anastomosis at a slightly lower position than usual to avoid tension in the ureter. In Case 2, without ureteral stent insertion, hydronephrosis was observed on the inner side kidney during the postoperative period. We believe that the absence of stenting resulted in anastomotic stricture and was one of the reasons for the eventual hydronephrosis. The graft bed space, ureter‐bladder anastomotic design, and wound closure were suspected to cause hydronephrosis; however, this could have been prevented if the stent had been placed. While stents of less common sizes, such as 3.0Fr and 3.7Fr, are commercially available, logistical planning is necessary to ensure their availability.

## Conclusion

We report two cases of EBKT from pediatric donors to teenage recipients. This report will help surgeons perform EBKT in the growing number of pediatric kidney donations, such as those in Japan.

## Author contributions

Takafumi Yagisawa: Writing—original draft. Taichi Kanzawa: Review and editing. Toshihito Hirai: Review and editing. Kohei Unagami: Review and editing. Yoko Shirai: Review and editing. Kiyonobu Ishizuka: Review and editing. Kenichiro Miura: Review and editing. Motoshi Hattori: Supervision. Hideki Ishida: Supervision. Toshio Takagi: Supervision.

## Conflict of interest

None declared.

## Approval of the research protocol by an Institutional Reviewer Board

Not applicable.

## Informed consent

We obtained an informed consent from the patients.

## Registry and the Registration No. of the study/trial

Not applicable.

## References

[1] Harambat J , van Stralen KJ , Schaefer F *et al*. Disparities in policies, practices and rates of pediatric kidney transplantation in Europe. Am. J. Transplant. 2013; 13: 2066–2074. 23718940 10.1111/ajt.12288

[2] Chesnaye NC , van Stralen KJ , Bonthuis M *et al*. The association of donor and recipient age with graft survival in paediatric renal transplant recipients in a European Society for Paediatric Nephrology/European Renal Association‐European Dialysis and Transplantation Association Registry study. Nephrol. Dial. Transplant. 2017; 32: 1949–1956. 28992338 10.1093/ndt/gfx261

[3] Yagisawa T , Mieno M , Ichimaru N *et al*. Trends of kidney transplantation in Japan in 2018: data from the kidney transplant registry. Ren. Replace. Ther. 2019; 5: 1–14.

[4] Sharma A , Fisher RA , Cotterell AH , King AL , Maluf DG , Posner MP . En bloc kidney transplantation from pediatric donors: comparable outcomes with living donor kidney transplantation. Transplantation 2011; 92: 564–569. 21869746 10.1097/TP.0b013e3182279107

[5] Winnicki E , Dharmar M , Tancredi D , Butani L . Comparable survival of en bloc versus standard donor kidney transplants in children. J. Pediatr. 2016; 173: 169–174. 26898807 10.1016/j.jpeds.2016.01.054

[6] Martin LW , Gonzalez LL , West CD , Swartz RA , Sutorius DJ . Homotransplantation of both kidneys from an anencephalic monster to a 17 pound boy with Eagle‐Barret syndrome. Surgery 1969; 66: 603–607. 4389959

[7] Thomusch O , Tittelbach‐Helmrich D , Meyer S , Drognitz O , Pisarski P . Twenty‐year graft survival and graft function analysis by a matched pair study between pediatric en bloc kidney and deceased adult donors grafts. Transplantation 2009; 88: 920–925. 19935464 10.1097/TP.0b013e3181b74e84

[8] Kayler LK , Magliocca J , Kim RD , Howard R , Schold JD . Single kidney transplantation from young pediatric donors in the United States. Am. J. Transplant. 2009; 9: 2745–2751. 20021480 10.1111/j.1600-6143.2009.02809.x

[9] Bhayana S , Kuo YF , Madan P *et al*. Pediatric en bloc kidney transplantation to adult recipients: more than suboptimal? Transplantation 2010; 90: 248–254. 20548262 10.1097/TP.0b013e3181e641f8

[10] Feltran Lde S , Nogueira PC , Silva FA *et al*. A one year prospective comparison of kidney growth and function in children recipients of grafts from children and adults. Transplantation 2010; 90: 777–781. 20689495 10.1097/TP.0b013e3181f009b7

